# A Woman's Experience in a Surgical Ward

**Published:** 1913-08-16

**Authors:** 


### HOSPITALS FROM THE PATIENTS' POINT OF VIEW.

(Contributions to this Section are Invited.)

### A Woman's Experience in a Surgical Ward.

The reluctance of patients to criticise their experiences while undergoing treatment in hospital is well illustrated by the difficulty we have found in inducing the young woman whose views are expressed in this article to contribute to the series. She declared that she felt so grateful to the hospital authorities, and so sensible of the kindness that had been shown her while in hospital, that she was quite averse to commenting on her experiences. She was evidently under the impression—an impression, be it noted, that is shared by most hospital patients —that to criticise hospital treatment is to be wholly ungrateful to the hospital staff and authorities, and to forget or ignore the help they had given during a time of great stress and trial. As we have already tried to point out, that view is not correct. Criticism, so long as it is informative, and given without animus, but with the intention of assisting the authorities by calling attention to remediable faults in the management of the institution, will always be welcomed. While we share our contributor's feeling that it is not wise to give the name of the institution concerned, we feel sure that hospitals will readily recognise that faults may exist, and that the authorities will be grateful for any attention called to these defects as well as for suggestions towards amending such faults. To those who are acquainted with our hospitals many of the faults noted in the present article will be familiar enough, and our contributor's experience is, therefore, in view of its outspokenness, singularly interesting.

The patient in this case was a young woman who was operated upon for an attack of acute suppurative appendicitis, complicated later on by pelvic peritonitis, which necessitated a further operation. The surgical measures needed for her treatment were severe, but no criticism is added with regard to this point since, being a lay person, her opinion would be of comparatively little value. She made a slow recovery, and was subsequently sent to a convalescent home. The notes from which the article has been drawn up were written at the home while the experiences were still fresh in the patient's mind.

### Ward Rules and "Temperatures."

She starts by gratefully commenting on the kindness of the staff. "The doctors are very attentive, putting in an appearance almost directly they are wanted. Thank goodness there are no troublesome ward rules. . . . They are very particular about the wards being kept scrupulously clean and the plants and flowers being taken out every night. Of course, I know that this is essential, but it seems to me that the nurses are not quite so careful in other things. For instance, when temperatures are taken the thermometer is passed from one patient to another. In some cases it is placed in the patient's mouth; in others under the arm. I can watch it passing down from one patient to another, until it comes to me—and I am supposed to have my temperature taken by mouth! The least they could do, surely, is to wash the thermometers after use in each case. But they don't seem to think of it."

She refers to small incidents which show that in this hospital there is a lack of discipline and careful supervision. This carelessness in the use of a thermometer illustrates one phase of hospital and ward neglect—a phase which is avoidable, since in most modern hospitals a separate thermometer is kept for each patient. The custom of passing the thermometer down from patient to patient is quite obsolete, while that of allowing the same thermometer to be used for mouth and axillary measurements, even on the same patient, cannot too severely be condemned.

"One day one of the nurses was to give me an enema. She brought a probationer to watch the process, but instead of showing the latter she allowed her to give the enema, with the result that the nozzle was inserted into the vagina." Here there was evidently gross carelessness, since so common an accident could, and ought, to have been remedied before the patient was aware of its occurrence.

### Sisters Wanting in Candour.

"The ward is very small, and there are only eight people in it. One of the patients is a small baby, only a few weeks old. It cries all day long and most of the night, and sometimes it is quite impossible for us to get any sleep owing to the noise it makes. . . . The nurses never think of closing a door behind them, so that besides the constant draught we have to endure there is a perpetual banging of doors, which jars all the beds."

The obvious suggestion here is that the doors should be made so that it is quite impossible for them to bang. In modern hospitals that is done; the doors are swing doors with rubber protectors in cases where they swing loosely, but usually they are made so well that they fit their frames and swing to and fro without making any jarring noise. To a patient who is lying in bed after a severe abdominal operation, especially to one who is suffering from peritonitis, the slightest shock is intensified and accentuated, and extra care should therefore be taken to protect the bed from such vibratory movement. In old wards with loose flooring and obsolete doors much may be done by putting a wedge of india-rubber under each leg of the bed to act as a buffer against the vibrations.

"I do not know what object they gain by doing so, but the ward sisters never seem to be truthful in answering the doctor's questions. One day I had a high temperature, and felt very ill so that I could not eat anything. This was known perfectly to sister, yet when the doctor asked her the next day if I was eating well she said, 'Very well, indeed.' The doctor also asked if the baby was ever sick after meals, and sister said, 'No, never.' As a matter of fact the baby was nearly always sick after it had taken its food." . . . Prevarication and the *suggestio falsi* are common enough in hospitals; the only effectual remedy against both is to be found in the strict enforcement of discipline by the staff and by the matron. A nurse who is found guilty of a "white lie" should be severely dealt with. The two instances mentioned by the patient were obviously of this nature; probably the sister thought that there was no need to worry the doctor since both the patient's and the baby's general condition were good. The neglect to report incidental lapses, however, is a dereliction of duty, and must be held to be such, no matter what excuse may be made.

Diet and Service.

In this institution the food was obviously, to judge from the patient's remarks, wholly unsuitable and badly prepared. We do not think that her statements are overdrawn or coloured in any way, and the facts she gives tend to show that the authorities should devote some attention to investigating the whole question of the feeding of convalescent patients at this institution. "It was a good thing my appetite was generally so bad, for I simply could not eat much of the food they supply here. There is never any salt in the dishes that need it, and not sufficient sugar in those that ought to be sweet. The food is always served either lukewarm or cold. We have fish twice a week; it is either boiled to a rag or is fried in bad fat and served up with cold potatoes, and an impossible stuff they call sauce. The meat we get is fearfully tough. Bread and butter is cut long before it is wanted, so that as a rule when we get it it is dry and chippy. . . The first time I was ordered beef-tea nurse brought me a feeder with mutton broth in it, and quite a layer of fat floating on top of the broth. Even the milk we get is not above suspicion. One night there was a slight thunderstorm, and the milk apparently turned, for the next day we had sour milk served with our tea. The only good milk in the ward that day was a small can specially kept in the larder which was destined for sister's breakfast. The milk is apparently never boiled in this hospital."

